# Adjuvant chemoradiotherapy after d2-lymphadenectomy for gastric cancer: the role of n-ratio in patient selection. results of a single cancer center

**DOI:** 10.1186/1748-717X-7-169

**Published:** 2012-10-15

**Authors:** Wilson L Costa, Felipe JF Coimbra, Ricardo C Fogaroli, Héber SC Ribeiro, Alessandro L Diniz, Maria Dirlei FL Begnami, Celso AL Mello, Marcelo F Fanelli, Milton JB Silva, José Humberto Fregnani, André L Montagnini

**Affiliations:** 1 Department of Abdominal Surgery, Hospital A. C. Camargo, Sao Paulo, Brazil; 2 Department of Radiotherapy, Hospital A. C. Camargo, Sao Paulo, Brazil; 3 Department of Surgical Pathology, Hospital A. C. Camargo, Sao Paulo, Brazil; 4 Department of Clinical Oncology, Hospital A. C. Camargo, Sao Paulo, Brazil; 5 Center for Researcher Support, Barretos Cancer Hospital, Barretos, Brazil; 6 Department of Gastroenterology, Faculty of Medicine, University of Sao Paulo, Sao Paulo, Brazil

## Abstract

**Background:**

Adjuvant chemoradiotherapy is part of a multimodality treatment approach in order to improve survival outcomes after surgery for gastric cancer. The aims of this study are to describe the results of gastrectomy and adjuvant chemoradiotherapy in patients treated in a single institution, and to identify prognostic factors that could determine which individuals would benefit from this treatment.

**Methods:**

This retrospective study included patients with pathologically confirmed gastric adenocarcinoma who underwent surgical treatment with curative intent in a single cancer center in Brazil, between 1998 and 2008. Among 327 patients treated in this period, 142 were selected. Exclusion criteria were distant metastatic disease (M1), T1N0 tumors, different multimodality treatments and tumors of the gastric stump. Another 10 individuals were lost to follow-up and there were 3 postoperative deaths. The role of several clinical and pathological variables as prognostic factors was determined.

**Results:**

D2-lymphadenectomy was performed in 90.8% of the patients, who had 5-year overall and disease-free survival of 58.9% and 55.7%. The interaction of N-category and N-ratio, extended resection and perineural invasion were independent prognostic factors for overall and disease-free survival. Adjuvant chemoradiotherapy was not associated with a significant improvement in survival. Patients with node-positive disease had improved survival with adjuvant chemoradiotherapy, especially when we grouped patients with N1 and N2 tumors and a higher N-ratio. These individuals had worse disease-free (30.3% vs. 48.9%) and overall survival (30.9% vs. 71.4%).

**Conclusion:**

N-category and N-ratio interaction, perineural invasion and extended resections were prognostic factors for survival in gastric cancer patients treated with D2-lymphadenectomy, but adjuvant chemoradiotherapy was not. There may be some benefit with this treatment in patients with node-positive disease and higher N-ratio.

## Introduction

Several Western studies have described a 20-30% 5-year survival for gastric cancer patients who are treated with curative intent [1], whereas in Asian countries surgery-only treated patients have reached 5-year survival near 60%-70% [2,3]. Lymph node dissection seems to have a major role in this difference, as individuals from Western centers where D2-lymphadenectomy is routinely performed have survival above 50% [4,5]. Even with optimal surgical treatment, gastric cancer relapse can be observed in over 40% of the patients [6].

The Intergroup US 0116 Trial (INT 0116) [7] demonstrated survival improvement with the addition of chemoradiotherapy after negative-margin resection of tumors without distant metastases. However, more than 50% of the patients had an incomplete D1 lymph node dissection, and among the 10% of individuals in whom a D2-lymphadenectomy was performed, no survival benefit was observed. For these patients, only one prospective non-randomized study demonstrated a gain of survival with adjuvant chemoradiotherapy [8]. The interpretation of this finding is limited by the retrospective analysis of the study and by conflicting results in the surgical group [9].

Toxicity is also a major concern in this set of multimodality treatment, as performance status is negatively impacted after gastroesophageal resection [10]. In INT 0116 [7], only 64% of the patients completed treatment protocol. Another retrospective series demonstrated a similar pattern of toxicity [11].

The aims of this study are to describe the results of gastrectomy with or without adjuvant chemoradiotherapy in gastric cancer patients treated in a single institution, and to identify prognostic factors that could help establish subgroups of patients who would benefit from this treatment.

## Materials and methods

### Patients

This is a retrospective study, which included patients with pathologically confirmed gastric adenocarcinoma who underwent surgical treatment with curative intent in a single cancer center in Brazil, in the period between September 1998 and December 2008. Tumor staging followed the AJCC/UICC TNM staging manual, 7^th^ edition [12], and lesions staged as IB through IIIC were included.

Exclusion criteria were: tumors of the gastric stump; any multimodality treatment different from the INT 0116 protocol [7], distant metastasis and T1N0 tumors. Patients lost to follow-up or who died postoperatively were excluded from the analysis.

In the period of our study, 327 patients had surgical resection for gastric cancer and 142 were analyzed. Among the excluded individuals, 69 had M1 disease, 50 had T1aN0 or T1bN0 tumors, 19 were diagnosed with tumors of the gastric stump, 34 received a different multimodality treatment, 10 were lost to follow-up and there were 3 postoperative deaths. The control group included patients consecutively treated with surgery only (90), most of them before 2005, and the other 52 were treated with resection followed by adjuvant chemoradiotherapy.

### Variables

Categories for the following clinicopathological variables were defined: age, gender, extent of gastric resection, type of lymphadenectomy, number of dissected nodes, extended gastrectomy with resection of adjacent organs, tumor site, Lauren`s histological type, T and N category (TNM 7^th^ ed. [12]), lymphatic vessel and perineural invasion.

The ratio between metastatic and dissected lymph nodes (N-ratio) was also assessed. The best cut-off intervals were based on the intervals described by Marchet A. et al. [13] (NR0 – 0%, NR1 – 1%-9%, NR2 – 10%-25%, N3 -> 25%). In this study, higher survival was identified in patients with N1 and N2 tumors and lower N-ratio. In the TNM 7^th^ ed., lesions previously classified as N1 were divided in N1 (1–2 positive nodes) and N2 (3–6 nodes). The interaction between N-ratio and N-category was established as described in a previous study [14]. With the new TNM staging [12], a new interaction was adopted, with the same intervals for N2 and N3 tumors and two new intervals for those who had N1 lesions (NR1a, with an N-ratio of 1-4% and NR1b, 5-9%), since all of them had N-ratio between 1 and 9%.

### Surgical treatment

Resection included a distal or total gastrectomy and D2-lymphadenectomy, as routinely recommended at our institution. A D1-dissection was only performed in patients who had poor performance status, in whom a more extended lymphadenectomy could represent additional operative time and higher morbidity. Pancreatectomy and splenectomy were only performed when deemed necessary so that negative margins be obtained.

### Adjuvant treatment

Gastric cancer patients treated with negative-margin resections and tumors staged as IB-IV M0 were started on adjuvant chemoradiotherapy (CT/RT) at our institution after 2004, according with the protocol described in the INT 0116 [7]. They should have had performance status of 2 or lower, adequate renal and liver function and an appropriate daily caloric intake. Treatment had to start no more than two months after surgery. All the patients were treated in 6 Mev Linear Accelerator with three-dimensional conformal therapy. Four fields (antero-posterior and two lateral fields) were used. Radiotherapy consisted of 45 Gy at 1,8 Gy per day, five days per week for five consecutive weeks, to tumor bed, regional nodes and 2 cm beyond the proximal and distal margins of resection. The tumor bed was defined by preoperative computed tomographic imaging.

### Survival outcomes

Overall survival was defined as the interval in months measured between the date of resection and death for any cause, or the date of the patient`s last appointment. Disease-free survival was determined as the period between the date of surgery and the relapse diagnosis, obtained by imaging tests or as an intraoperative finding, or death, whichever happened first.

### Statistical analysis

For statistical analysis, the software “Statistical Package for Social Science” (SPSS), version 15.0 was used. The quantitative variables were expressed by the measure of appropriate central tendency (mean or median) and respective measure of variability. The comparison of the two groups regarding their clinicopathological characteristics was performed by using Student`s t test or Mann–Whitney test for quantitative data. For qualitative variables, Pearson`s chi-square or Fisher`s exact test were used. The analysis of overall and disease-free survival was done by the estimator product-limit of Kaplan-Meier and the comparison of curves was done through log-rank test. The variables that statistically had p<0.20 for the test of log-rank were selected for the multiple analysis using the Cox proportional hazards model.

## Results

### Patients and surgical treatment

Among the 142 patients in the study, 81 were male and their median age was 63 years, ranging from 21 to 88.

Regarding resection, 80 individuals had a total gastrectomy, and the other 62 had a distal gastrectomy. D2-lymphadenectomy was performed in 129 patients, while the other 13 had a more limited lymph node dissection because of poor performance status. The median number of dissected nodes was 34 (10–84). An extended gastrectomy was deemed necessary in 26 individuals, with 22 having splenectomy, 5 distal pancreatectomy, 3 colectomy and 1 left lateral liver resection for locally advanced disease. Median operative time was 360 minutes (180–840), and only 19 patients had blood transfusion.

Overall morbidity was 24.6%, including minor and major postoperative events. The most common events were pneumonia in 8 patients, intraabdominal abscess in 5, superficial surgical site infection in 5, pancreatic leaking and central line infection in 3 individuals. Median time of hospital stay was 10 days (6–72). Mortality up to 90 days after surgery was 2.4% (3 cases: 1 due to pneumonia, 1 due to complications related to esophagojejunal anastomosis dehiscence and another for pulmonary thromboembolism).

### Pathology findings

The gastric tumors were most frequently located in the body and antrum (59 and 60 cases, respectively). Regarding Lauren histological type, 74 patients had intestinal tumors, and 68 had diffuse ones. Tumors invading serosa occurred in 85 individuals, and 101 had lymph node positive disease. The presence of lymphatic vessel invasion was observed in 69 patients and 64 had perineural invasion. N-ratio distribution was uniform, with slightly higher number of NR0 and NR1 patients.

Clinicopathological features are presented in Table 1, along with the survival outcomes of the studied population.

**Table 1:** Clinicopathological characteristics and prognostic factors in overall and disease-free survival of patients treated for gastric cancer

**Variable**	**No. patients (n=142)**	**Overall survival (5-yr)**	**P**	**Disease-free survival (5-yr)**	**P**
**Gender**					
Male	81	58.7%	0.704	54.1%	0.645
Female	61	58.9%		57.7%	
**Age**					
40 years	10	70.0%	0.321	60.0%	0.450
41-69 years	86	62.9%		59.7%	
≥ 70 years	46	49.6%		47.5%	
**Gastrectomy**					
Total	80	49.7%	0.024	43.9%	0.007
Subtotal	62	70.4%		70.4%	
**Extended resection**					
Yes	26	35.5%	0.004	34.6%	0.002
No	116	64.3%		60.6%	
**Lymphadenectomy**					
D1	13	50.5%	0.270	47.8%	0.388
D2	129	59.9%		56.3%	
**No. of dissected nodes**					
Less than 25	39	54.3%	0.339	48.8%	0.269
25 or more	103	60.6%		58.0%	
**Location**					
Cardia	21	37.0%	<0.001	27.7%	<0.001
Body	59	60.3%		57.9%	
Antrum	60	66.8%		64.4%	
Linitis	2	0%		0%	
**Lauren histology**					
Intestinal	74	61.4%	0.580	57.1%	0.748
Diffuse	68	55.6%		54.1%	
**Lymphatic vessel invasion**					
Yes	59	48.8%	0.032	44.7%	0.023
No	83	66.7%		63.8%	
**Perineural invasion**					
Yes	67	48.4%	0.015	42.2%	0.003
No	75	68.9%		68.3%	
**T-category**					
T1	11	68.6%	0.005	68.6%	<0.001
T2	29	78.4%		71.5%	
T3	11	61.4%		61.4%	
T4a	85	53.8%		51.2%	
T4b	6	33.3%		16.7%	
**N-category**					
N0	40	75.8%	0.002	75.8%	0.005
N1	24	63.8%		57.8%	
N2	42	57.7%		53.6%	
N3a	29	43.4%		39.3%	
N3b	7	14.3%		14.3%	
**N-ratio**					
NR0	40	75.8%	0.005	75.8%	0.001
NR1	39	69.8%		66.3%	
NR2	27	43.7%		39.9%	
NR3	26	36.1%		31.0%	
**N-category/N-ratio interaction**					
N0-NR0	40	75.8%	0.002	75.8%	0.001
N1-NR1a	10	76.2%		76.2%	
N1-NR1b	14	57.1%		45.7%	
N2-NR1	15	79.0%		79.0%	
N2-NR2 and NR3	27	45.1%		39.5%	
N3a-NR2 and NR3	29	43.4%		39.3%	
N3b-NR3	7	14.3%		14.3%	

### Groups comparison

The group of patients treated with surgery-only, which was used as a historical control, was compared with those who received adjuvant chemoradiotherapy. They were similar when compared by gender, extent of resection, lymphadenectomy, extended gastrectomy, tumor site and Lauren histology, number of dissected nodes, lymphatic vascular invasion, perineural invasion and T category.

Patients treated with chemoradiotherapy were younger, with a median of 55 years old, while the surgical group had a median of 63 years (P<0.001). There was also a significant difference regarding N-category, as 40% of the surgery-only patients had N0 disease, whereas there were only 5 N0 cases (9.6%) in the chemoradiotherapy group (P>0.001).

### Adjuvant therapy

Among the 52 individuals treated with adjuvant chemoradiotherapy, 40 completed treatment (76.9%). Regarding toxicity, 20 patients (38%) had grade II and 8 had grade III/IV gastrointestinal toxicity, while 19% of them developed grade III/IV hematological toxicity. Two adjuvant-related treatment deaths were observed; one patient died from hematological toxicity while having chemotherapy associated with radiotherapy, and the other had a late colonic perforation due to actinic effects, and died after surgery.

### Survival outcomes

The population of the study had a median follow-up of 45 months (49 months for the surgical group and 37 for the adjuvant CT/RT one). Those who were alive with or without recurrence had median follow-up of 56 and 69 months, respectively. Estimated 5-year overall survival was 58.9%, and 5-year disease-free survival was 55.7%.

Relapse occurred in 47 patients (33.6%), 32 in the surgery-only group (35.5%) and 15 in the adjuvant treatment one (28.8%). The first documented site of recurrence was loco-regional in 23.9%, liver in 30.4%, peritoneal in 32.6% and other distant sites in 13%. Patients who received chemoradiotherapy had lower loco-regional recurrence (21.4% vs. 25%) and a slightly higher systemic relapse (78.6% vs. 75%), although this difference was not statistically significant.

The factors that influenced overall and disease-free survival included: type of gastrectomy, tumor site, extended resection, lymphatic vessel and perineural invasion, N-ratio, T-category and N-category (Table 1).

**Figure 1:**
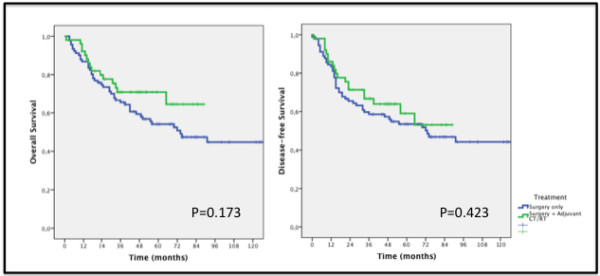
Overall and Disease-free survival of gastric cancer patients treated with surgery with or without adjuvant chemoradiotherapy.

Patients who received adjuvant chemoradiotherapy had 5-year overall survival of 70.9% and disease-free survival of 59.1%. They had better numbers than the surgery-only group, which were respectively 54.1% and 53.5%. These results were not statistically significant though (Figure 1).

For multivariate analysis, the interaction between N-ratio and N-category was established as described in a previous study [14] and with the following categories: N0-NR0; N1-NR1a; N1-NR1b; N2-NR1; N2-NR2 and NR3; N3a-NR2 and NR3; N3b-NR3. It was an independent prognostic factor for overall and disease-free survival, along with extended gastrectomy and perineural invasion (Table 2).

**Table 2:** Multivariate analysis prognostic factors in overall and disease-free survival of patients treated for gastric cancer

**Variable**	**Overall survival**		**Disease-free survival**	
	**HR (CI 95%)**	**P**	**HR (CI 95%)**	**P**
**Perineural invasion**				
No	1.0		1.0	
Yes	2.03 (1.17-3.53)	0.011	2.46 (1.46-4.15)	0.00
**Extended resection**				
No	1.0		1.0	
Yes	2.40 (1.31-4.38)	0.004	2.58 (1.46-4,56)	0.001
**Treatment modality**				
Surgery only	1.61 (0.85-3.04)	0.142	*	*
Adjuvant CT/RT	1.0		*	*
**N-category – N-ratio**				
N0-NR0	1.0	0.021	1.0	0.008
N1-NR1a	0.72 (0.16-3.17)	0.661	0.65 (0.15-2.86)	0.568
N1-NR1b	1.92 (0.73-5.05)	0.184	2.01 (0.81-4.96)	0.131
N2-NR1	0.51 (0.14-1.81)	0.297	0.41 (0.20-1.44)	0.167
N2-NR2 and NR3	2.02 (0.93-4.39)	0.076	2.26 (1.10-4.63)	0.026
N3a – NR2 and NR3	2.78 (1.31-5.89)	0.008	2.66 (1.30-5.44)	0.007
N3b – NR3	2.96 (1.08-8.12)	0.036	2.77 (1.03-7.44)	0.043

### The role of adjuvant chemoradiotherapy in survival outcomes

In order to identify a specific group of patients who would benefit from this set of adjuvant treatment, its influence in survival for all categories of every prognostic factor identified in univariate analysis was tested. Patients who had lymph node metastasis had a significant improvement in overall survival (P=0.023) with the addition of adjuvant chemoradiotherapy. The same benefit was not observed in any other category (Table 3).

**Table 3:** Survival outcomes stratified by prognostic factors in overall and disease-free survival of patients treated for gastric cancer with or without adjuvant chemoradiotherapy

**Variable**	**No. patients**		**Overall survival**		**P**	**Disease-free survival**		**P**
	**Surgery only (n=90)**	**Adjuvant CT/RT (n=52)**	**Surgery only**	**Adjuvant CT/RT**		**Surgery only**	**Surgery CT/RT**	
**Lymphatic Vessel Invasion**								
Yes	39	20	43.1%	65.8%	0.198	39.5%	41.7%	0.567
No	51	32	62.3%	76.8%	0.458	61.6%	68.8%	0.735
**Perineural Invasion**								
Yes	47	20	43.7%	62.9%	0.470	40.7%	42.8%	0.787
No	43	32	64.0%	79.2%	0.222	63.1%	71.7%	0.603
**Extended resection**								
Yes	18	8	33.3%	45.0%	0.546	33.3%	37.5%	0.951
No	72	44	59.0%	76.2%	0.219	58.5%	63.4%	0.486
**Lymph node metastases**								
Yes	55	46	40.6%	68.2%	0.023	40.6%	53.7%	0.113
No	35	6	73.4%	100.0%	0.736	73.4%	50.0%	0.716
**T-category**								
T1	4	7	75.0%	66.7%	0.627	75.0%	66.7%	0.627
T2	21	8	70.6%	100.0%	0.096	70.6%	71.4%	0.629
T3	5	6	25.0%	85.7%	0.081	25.0%	83.3%	0.170
T4a	55	30	51.3%	62.0%	0.875	50.1%	52.4%	0.999
T4b	5	1	20.0%	100.0%	0.259	20.0%	0.0%	0.953
**N-category**								
N0	35	5	73.4%	100.0%	0.736	72.4%	100.0%	0.689
N1	14	10	50.0%	80.0%	0.203	50.0%	52.5%	0.674
N2	22	20	46.5%	73.1%	0.140	45.0%	64.3%	0.279
N3a	14	15	32.7%	58.7%	0.340	32.7%	41.7%	0.439
N3b	5	2	16.7%	0.0%	0.758	16.7%	0.0%	0.919
**N-category – N-ratio**								
N0 – NR0	35	5	73.4%	100.0%	0.736	72.4%	100.0%	0.689
N1 – NR1a	5	5	80.0%	75.0%	0.688	80.0%	75.0%	0.688
N1 – NR1b	9	5	37.5%	83.3%	0.106	37.5%	40.0%	0.553
N2 – NR1	8	7	75.0%	83.3%	0.620	75.0%	85.7%	0.656
N2 – NR2 and NR3	14	13	27.8%	67.7%	0.120	26.8%	53.8%	0.251
N3a – NR2 and NR3	14	15	32.7%	58.7%	0.340	32.7%	41.7%	0.439
N3b – NR3	5	2	16.7%	0.0%	0.758	16.7%	0.0%	0.919

The role of this multimodality treatment was then tested in different groups of patients who had lymph node metastasis, by using the N-category – N-ratio interaction. Patients with N1 and N2 tumors and higher N-ratio had similarly superior survival numbers with the addition of chemoradiotherapy (Table 3), but this data was not statistically significant. These 65 individuals were then divided in two groups, one including 25 patients with N1-NR1a or N2-NR1 disease (Group 1), and 40 with N1-NR1b or N2-NR2 and NR3 disease (Group 2). No statistical differences between the groups were observed (Table 4). The individuals in Group 1, with a lower N-ratio, had no benefit with adjuvant chemoradiotherapy both in overall (78.8% vs. 76.9%) and disease-free survival (81.8% vs. 76.9%), whereas those in Group 2 who had adjuvant treatment had a significant improvement in overall survival (71.4% vs. 30.9%; P=0.038) and superior but not statistically significant numbers regarding disease-free survival (48.9% vs. 30.3%; P=0.145).

**Table 4:** Clinicopathological characteristics of the two N-category / N-ratio groups determined

**Variable**	**Group 1 (N1-NR1a N2-NR1)**	**Group 2 (N1-NR1b N2-NR2 and NR3)**	**P**
**Gender**			
Male	10	25	0.077
Female	15	15	
**Age**			
Mean	60,1	64,2	0.249
**Gastrectomy**			
Total	14	23	0.905
Subtotal	11	17	
**Extended resection**			
Yes	21	32	0.477
No	4	8	
**No. of dissected nodes**			
Median	40 (23–69)	28 (10–50)	<0.001
**Location**			
Cardia	1	9	0.130
Body	11	15	
Antrum	13	16	
**Lauren histology**			
Intestinal	12	21	0.724
Diffuse	13	19	
**Lymphatic vessel invasion**			
Yes	9	16	0.747
No	16	24	
**Perineural invasion**			
Yes	12	15	0.403
No	13	25	
**T category**			
T1	5	2	0.286
T2	4	8	
T3	3	3	
T4a	11	25	
T4b	2	2	

## Discussion

Adjuvant chemoradiotherapy after complete resection for gastric cancer was associated with an improvement in overall and disease-free survival [7] and has been adopted in many Western centers since [15]. Some issues should be addressed though, surgical control being the major one. In the INT0116, D2-lymphadenectomy was recommended as part of the surgical treatment. However, it was performed in only 10% of the patients, who had a median survival of 48 months and no improvement with adjuvant treatment, whereas the whole study population had median survival of 36 months [7]. The use of this treatment in patients who had D2-lymphadenectomy was first reported in a nonrandomized Korean study with 890 patients, in which adjuvant treatment was associated with an improvement in survival (57% vs. 51%). This same group led the just published ARTIST Trial, a phase III study that compared the adjuvant treatment with chemotherapy (6 cycles of XP - Capecitabine and Cisplatin) vs. the association of chemotherapy and radiotherapy (2 cycles of XP + XP and Radiotherapy + 2 more cycles of XP) after D2-lymphadenectomy. The addition of radiotherapy provided an improvement in disease-free survival for node-positive patients [16].

Patterns of relapse are another point of concern. In the INT 0116 trial, adjuvant chemoradiotherapy was associated with a decrease in local recurrence (29% vs. 19%), but not in systemic/peritoneal relapse. A large Eastern series with 2328 patients described loco-regional relapse to be around 20% with surgery only [17]. Even Western studies demonstrate that when D2-lymphadenectomy is performed less than 20% of the patients develop loco-regional recurrence [18].

Debates remain among some Western authors regarding the extent of lymphadenectomy for gastric cancer. They are based in the negative results of two randomized trials published in 1999. Their results should be interpreted with caution, mainly due to the adverse effect of D2-related pancreatic and splenic resections in postoperative mortality. A recent update in the results of one of these trials should put an end to this controversy, as D2-lymphadenectomy was associated with a significant improvement in cancer-related mortality and in loco-regional recurrence [19].

The present study included 90.8% of patients who were treated with D2 dissection. Severe postoperative morbidity was low and postoperative deaths were excluded, which suggests that the number of patients who were programmed to have adjuvant chemoradiotherapy and did not receive it was very low. The treatment was not associated with significant improvement in overall and disease-free survival. Some limitations about this finding should be discussed. The first one regards the two differences between the group of patients treated with resection, and the ones treated with adjuvant chemoradiotherapy, who were younger and had higher frequency of node-positive tumors. In this case, treatment could offset a worse result in patients with node-positive disease. Another limitation of the study resides in the fact that this is a retrospective cohort of consecutive cases and the more recently treated individuals were the ones who had adjuvant chemoradiotherapy and, therefore, have a shorter follow-up. The use of a historic control may represent another bias, albeit the low morbidity and mortality of patients throughout the study and the fact that all the patients were operated by the same group of surgeons.

Based on the conflicting results in the literature on the subject of chemoradiotherapy in patients treated with D2-lymphadenectomy, the toxicity it is associated with, and this own series numbers, which showed better but not significant results with adjuvant treatment, the goal was to try to identify prognostic factors that could establish which individuals would benefit from adjuvant treatment.

Besides extended resections, perineural invasion was also an independent prognostic factor associated with worse survival, as has been shown in the literature, especially if associated with lymphatic vessel invasion and early tumors [20].

However, the most important prognostic factor for overall and disease-free survival was the interaction between N-category and N-ratio. It has been shown that this interaction individualizes groups of patients with distinguished survival numbers within the same N-category in TNM staging 6^th^ edition [14], confirming the finding in other studies that N-ratio could determine different outcomes in patients with N1 and N2 tumors [13,21]. The role of this interaction was now investigated in the new TNM staging system 7^th^ edition [12] and again different survival outcomes were identified in patients with N1 and N2 tumors (N2-NR2 and NR3 tumors – HR 2.02 for death and 2.26 for recurrence).

When analyzing the role of adjuvant chemoradiotherapy for each variable category, an improvement in overall survival among patients with node-positive disease was observed. However, by studying the distribution of these patients in different N-category N-ratio categories, individuals with N1 and N2 lesions and higher N-ratio seemed to have better survival with adjuvant chemoradiotherapy. By grouping these patients, this difference in outcomes was more easily identified, with a significant improvement in overall and better numbers albeit not statistically significant in disease-free survival. Those who had D2-lymphadenectomy with higher N-ratio had the worse outcomes, possibly because residual node disease was left behind. To measure the likelihood of disease in undisected regional nodes, the use of the Maruyama index (MI) has already been described. An MI < 5 was an independent survival prognostic factor among the INT0116 patients and it proved to be a valuable surgical undertreatment detection tool [22].

The most important limitation in our study refers to the small number of patients, especially in the subgroups of N1/N2 tumors, with 65 in total. This may provide a low statistical power for the results. However, even in this small subset, very different numbers were observed and a statistically significant result was obtained, which favored the rationale of using N-ratio associated with N-category to help identify patients who supposedly had a D2-lymphadenectomy and would benefit from adjuvant chemoradiotherapy. This interaction worked as a numeric criterion that might represent, after further studies, an absolute, reproducible factor that could help select patients for this treatment could have been established.

In conclusion, in this single center series, adjuvant chemoradiotherapy was not a prognostic factor for overall and disease-free survival in gastric cancer patients treated with D2-lymphadenectomy. Patients with N1 and N2 tumors and higher N-ratios could benefit from this multidisciplinary treatment.

## Competing interests

All the authors report no competing interest**.**

## Authors’ contributions

WLC Jr, FJFC, RCF, HSCR, ALD, MDFSB, CALM, MFF, MJBS, JHF, ALM. All authors read and approved the manuscript.
